# Managing Gut Dysbiosis: Clinical Evidence and Perspectives on *Saccharomyces boulardii* CNCM I-745 for Diarrhoeal Conditions in Adults and Children

**DOI:** 10.3390/microorganisms14010150

**Published:** 2026-01-09

**Authors:** Gerardo Pio Nardone, Arrigo Francesco Giuseppe Cicero, Luca Gallelli, Roberto Berni Canani

**Affiliations:** 1 Department of Clinical Medicine and Surgery, University of Naples “Federico II”, 80131 Naples, Italy; 2 Department of Medical and Surgical Sciences, Alma Mater Studiorum—University of Bologna, 40126 Bologna, Italy; arrigo.cicero@unibo.it; 3 Department of Health Sciences, University “Magna Graecia” of Catanzaro, 88100 Catanzaro, Italy; gallelli@unicz.it; 4 Department of Translational Medical Science and NutriTechLab, University of Naples “Federico II”, 80131 Naples, Italy; berni@unina.it

**Keywords:** dysbiosis, probiotics, *Saccharomyces boulardii*, paediatrics, adults, gastrointestinal disorders, antibiotic-associated diarrhoea

## Abstract

The human gut microbiota plays a key role in health and disease across the lifespan and is shaped by complex intrinsic and extrinsic factors. Dysbiosis is increasingly recognized as a contributor to a wide range of clinical conditions, with diarrhoea—particularly antibiotic-associated diarrhoea—representing an early clinical marker of microbiota disruption. This narrative review summarizes current evidence on the probiotic yeast *Saccharomyces boulardii* CNCM I-745 and its clinical applications in both paediatric and adult populations. Available clinical data support its safety and efficacy in the prevention and management of gastrointestinal disorders, particularly diarrhoeal conditions, and suggest a potential role in promoting microbiota resilience. Key mechanisms of action, safety considerations, and findings from randomized controlled trials and meta-analyses are discussed. However, current data remains limited by heterogeneity among studies and a lack of long-term, mechanistic data, highlighting the need for further well-designed studies to clarify its role across different clinical settings.

## 1. Introduction

Over the past two decades, research on the human microbiota has experienced significant growth, attracting increasing attention not only within the scientific community but also among the public. This heightened attention stems from the recognition of the microbiota’s potential for medical applications, encompassing preventive measures and new therapeutic strategies across a diverse array of diseases [1].

The human microbiota has emerged as a crucial factor in health and disease, often conceptualized as the body’s “last organ” due to its vast genetic and metabolic capabilities [1,2]. A 70 kg adult is estimated to harbour approximately 3.8 × 10^13^ bacterial cells, a number comparable to human cells, forming a highly integrated ecosystem shaped by hundreds of millions of years of host–microbe co-evolution [2]. The gut microbiome encompasses not only bacteria but also archaea, other eukaryotic microorganisms and fungi [1]. The gut “mycobiome”, though representing only a small fraction of the intestinal microbiota (approximately 0.01–0.1%), plays a relevant role in host health and immunity due to the distinct immunoregulatory properties of fungal cells. Factors such as diet, antimicrobial exposure, and age can disrupt fungal composition, contributing to dysbiosis and intestinal inflammation. Interactions between gut fungi, bacteria, and the host immune system are therefore central to intestinal homeostasis, with fungal components involved in immune pathways including Th17 cell activation [3]. Although fungi are now widely recognized as integral members of the microbiome, questions remain regarding the inclusion of other components such as phages, viruses, and mobile genetic elements [1].

Within this evolving framework, the gut microbiome is increasingly appreciated as a complex ecosystem that contributes to host development, metabolic regulation, and physiological homeostasis through essential microbial activities, including nutrient processing, fibre fermentation, neurotransmitter production, and the synthesis of vitamins and short-chain fatty acids [1,4,5,6,7]. Furthermore, the gut microbiota provides defence against pathogens, strengthens mucosal barrier integrity, and modulates local and systemic immune responses [5,6,7].

Through these mechanisms, microbial signals extend beyond the intestine, mediating interconnected “gut-organ axes” that impact the brain, liver, lungs, kidneys, and systemic immunity [6]. Generally, gut dysbiosis is characterised by diminished microbial diversity, a reduction in beneficial or keystone microbes within the core microbiota, and the proliferation of opportunistic pathogens [8,9]. Dysbiosis can manifest as disturbances in both structural composition and the functional performance of the gut microbiome. While some of these changes may be temporary and reversible, others can become persistent and irreversible. The outcome of these changes is influenced by the nature of the perturbation as well as by the baseline composition and functionality of the gut microbiota and host-related determinants [10,11,12]. When these alterations become irreversible, they may adversely impact host health, contributing to impaired gut barrier function and a broad spectrum of conditions, including gastrointestinal, hepatic, renal, metabolic, cardiovascular, and neurological disorders, as well as autoimmune diseases, diabetes, and liver cirrhosis [11,13,14]. Additionally, dysbiosis has been correlated with oxidative stress, elevated intestinal permeability, and systemic immune dysregulation [7,15].

Among the major drivers of gut dysbiosis, prolonged and repeated antibiotic treatment plays a central role. Antibiotic use profoundly influences gut microbiota composition and function, with evidence suggesting that its effects may range from transient perturbations to long-lasting alterations, potentially impacting microbiota resilience depending on the duration, frequency, and context of therapy [12]. Antibiotic exposure induces several alterations in gut microbiota, leading to dysbiosis characterized by loss of beneficial taxa, reduced microbial diversity, and overgrowth of opportunistic microorganisms. These changes promote intestinal inflammation, impair fluid and nutrient absorption, and contribute to the development of antibiotic-associated diarrhoea (AAD) [15].

Restoring microbial equilibrium has become a primary therapeutic objective, with probiotics—defined as “live microorganisms that, when administered in adequate amounts, confer a health benefit on the host”, being among the most thoroughly researched approaches [16,17].

This narrative review aims to provide a comprehensive overview of the clinical implications of gut dysbiosis, with a particular focus on the probiotic *Saccharomyces boulardii* CNCM I-745 (*S. boulardii*). By summarizing current evidence from both paediatric and adult populations, this review evaluates the role of *S. boulardii* in the prevention and management of gastrointestinal disorders in which dysbiosis contributes to disease pathogenesis, such as AAD. In this context, the efficacy and safety of *S. boulardii* as an adjuvant to *Helicobacter (H.) pylori* eradication therapies are also examined.

## 2. Probiotics: Definition, Mechanisms of Action, and Regulation

The gut microbiota accounts for approximately 0.2–2 kg of body weight and is predominantly localised in the colon, where microbial density reaches about 10^11^ to 10^12^ organisms per millilitre [18]. Alterations to the intestinal microbiota induced by dysbiosis significantly affect the host’s metabolic equilibrium. To facilitate the restoration of the gut microbiome after dysbiosis, or to promote a healthy microbial balance, several exogenous interventions have been proposed to enhance its resilience. Among these, probiotics represent a well-established strategy [19]. Probiotics are live microorganisms that, when administered in adequate amounts, confer measurable health benefits by promoting gut microbiota homeostasis and limiting the production of deleterious metabolites from pathogenic species [16].

Probiotics exert their effects through multiple mechanisms, including modulation of environmental pH, production of antimicrobial compound such as bacteriocins, competition with pathogens for essential nutrients, and stimulation of host immune responses [4]. In addition to limiting microbial overgrowth and preventing the onset of dysbiosis, probiotics provide further advantages These include the fermentation of non-digestible fibres to produce short-chain fatty acids (SCFAs), the biosynthesis of vitamins and metabolic cofactors, the promotion of anti-inflammatory cytokines and T-cell activity, and the strengthening of intestinal barrier integrity via the upregulation of tight junction proteins. Moreover, by sustaining microbial diversity, functional redundancy, and metabolic flexibility, probiotics enhance the resilience and long- term stability of the gut ecosystem [6]. Evidence suggests that the timely administration of an appropriate dosage of probiotics, either at the initiation of antibiotic therapy or within 48 h, can prevent or reduce the consequences of antibiotic-associated dysbiosis, such as diarrhoea, by supporting gut microbiota resilience and facilitating a return to the pre-antibiotic state [20].

The field of probiotics is experiencing rapid growth; however, several investigations have raised concerns regarding both the microbiological quality and the accuracy of labelling information on many probiotic products [21,22]. For probiotic formulations to confer significant health benefits, they must contain an adequate quantity of well-characterised microbial species and strains, ensuring their viability throughout transit within the gastrointestinal tract [23]. International guidelines consistently emphasise the need for rigorous quality control of probiotic products, including accurate strain identification and confirmation of microbial viability [16,24].

The regulatory framework governing probiotics varies widely across countries and depends on their classification as drugs, dietary supplements, or functional foods, resulting in substantial differences in approval requirements, quality standards, and clinical validation. Probiotic drugs are subject to stringent pre- and post-marketing controls, whereas dietary supplements and functional foods follow less rigorous regulatory pathways. Overall, this regulatory heterogeneity contributes to significant variability in the compositional quality and reliability of commercially available probiotic formulations [23].

European Society for Paediatric Gastroenterology, Hepatology and Nutrition (ESPGHAN) and Food and Agriculture Organization of the United Nations/World Health Organization (FAO/WHO) recommend clear product labelling that specifies the genus, species, strain, and the minimum number of viable microorganisms guaranteed at the end of shelf life [16,24].

Within the Italian regulatory framework, extensive experience in the field, together with the considerable number and complexity of probiotic preparations available, has resulted in stricter requirements compared with other European Union nations. The Italian Guidelines on Probiotics and Prebiotics, initially issued by the Ministry of Health in 2011 and periodically updated, establish explicit criteria to ensure the quality of probiotic products, including safety standards designed to protect consumers. The most recent update, issued in March 2018 [25], delineates the criteria for probiotics designated for human use, such as species- and strain-level identification, the minimum daily dose of viable microorganisms, and the permissible variance between the declared viable cell count on the label and that measured at the conclusion of the product’s shelf life. The effectiveness of probiotic interventions largely depends on the use of high-quality, well-characterized strains [26]. A critical aspect is that probiotic effects are strain-specific, with efficacy influenced by host-related factors such as age, baseline microbiota composition, and environmental conditions; therefore, every strain included in a commercial product must be explicitly validated in its clinical context, as data from one strain cannot be extrapolated to others.

In this context, the use of probiotic formulations registered as medicinal products may provide additional assurances of both strain effectiveness and product quality, owing to their specific and stringent regulatory pathway, distinct from that of dietary supplements or medical devices. Although, extensive research has focused on bacterial probiotics, studies concerning yeast-derived probiotics remain comparatively limited.

**Figure 1:**
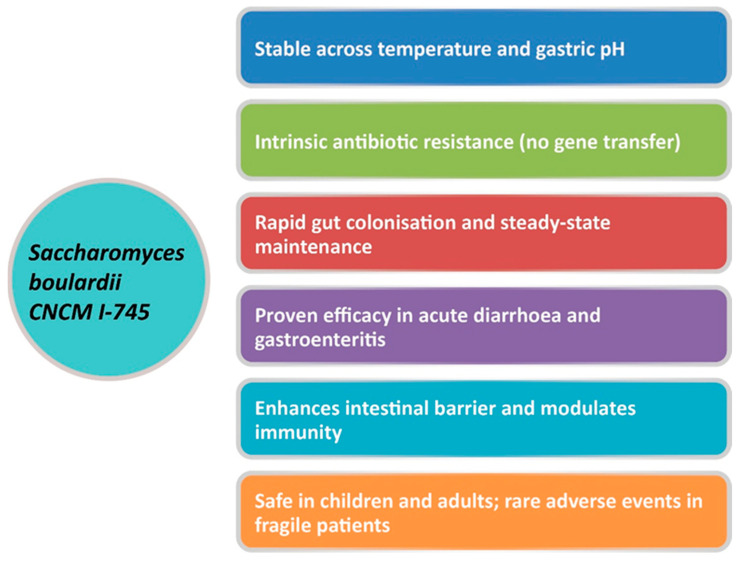
Unique properties of *S. boulardii* CNCM I-745. Adapted from Gopalan et al., 2023 [27].

Yeasts naturally possess resistance to antibacterial agents, presenting a distinct advantage as probiotic candidates. *S. boulardii* CNCM I-745 is a probiotic yeast belonging to the Saccharomyces cerevisiae species, originally identified by Henri Boulard in 1923 [27]. Among yeasts, *S. boulardii* stands out for its diverse beneficial properties and long-standing clinical use [28]. This non-pathogenic yeast was the first non-bacterial strain to be systematically investigated and applied as a human probiotic, with well-documented efficacy and safety in clinical practice [26]. It differs substantially from bacterial probiotics in terms of size, cell wall architecture, and metabolic functions. Importantly, it is intrinsically resistant to all antibiotics and does not acquire resistance genes, thus allowing its simultaneous administration during antibiotic treatment while maintaining full viability [29]. Pharmacokinetically, it withstands gastric acidity and bile salts, proliferates at 37 °C, reaches a steady state in the intestine within approximately three days, and is eliminated within 3–5 days after discontinuation [26]. *S. boulardii* employs multiple mechanisms of action, categorized into luminal, trophic, and mucosal- anti-inflammatory signaling effects [30]. Within the intestinal lumen, *S. boulardii* can interfere with pathogenic toxins and their adherence, interact with the resident microbiota, maintain cellular physiology, and restore short-chain fatty acid levels. Furthermore, *S. boulardii* may modulate the immune system, both locally within the lumen and systemically [26,30,31,32]. The key features of *S. boulardii* CNCM I-745 are summarized in Figure 1 [27].

**Figure 2:**
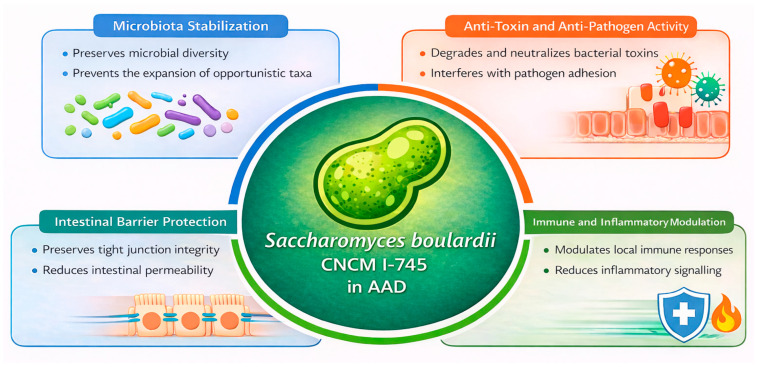
Biological Mechanisms Supporting the Use of *Saccharomyces boulardii* CNCM I-745 in AAD. Adapted from Terciolo et al., 2019 [31] and Czerucka D et al., 2019 [32]. Antibiotic-Associated Diarrhoea (AAD).

Clinical evidence supports the efficacy of *S. boulardii* CNCM I-745 in several gastrointestinal disorders, and it is increasingly considered a useful adjunct to antimicrobial therapy. This probiotic has demonstrated both preventive and therapeutic benefits, particularly in AAD, by reducing diarrhoeal incidence and duration [27,28,29,30,31,32]. These clinical effects are supported by multiple mechanisms of action. During antibiotic exposure, *S. boulardii* CNCM I-745 helps preserve microbiota stability by limiting antibiotic-induced shifts in microbial composition and preventing the expansion of opportunistic taxa [31]. In parallel, it exerts direct anti-toxin activity, including degradation and neutralisation of bacterial toxins, and interferes with pathogen adhesion to the intestinal epithelium [32]. At the mucosal level, *S. boulardii* reinforces epithelial barrier integrity by protecting tight junctions and reducing intestinal permeability, while also modulating local immune responses and inflammatory signalling pathways (Figure 2) [31,32]. Owing to this multifaceted mode of action, together with its favourable safety profile and extensive clinical validation, *S. boulardii* CNCM I-745 is widely regarded as a preferred probiotic option for the prevention and management of AAD and paediatric acute gastroenteritis (PAGE) [27].

## 3. Paediatric Gut Dysbiosis and the Role of *Saccharomyces boulardii*

The gut microbiota contributes substantially to human health across all stages of life, with its influence being especially pronounced during infancy [33]. The establishment of the infant gut microbiota progresses rapidly during the first months of life, driven by maternal and environmental microbial inputs, and typically reaches a relatively stable configuration by around three years of age [34,35]. However, recent evidence indicates that the maturation of the gut microbiome can extend further, reaching completion only around six years of age in humans [36,37]. During this trajectory, the infant gut microbiota undergoes a stepwise transformation, progressively acquiring the diversity and functional complexity that characterise the adult ecosystem [38]. This developmental period has been defined as a “critical window”, during which the intestinal community remains highly plastic and particularly vulnerable to disruptions such as dysbiosis [39].

Early-life exposure to antibiotics, whether during pregnancy, delivery, lactation, or directly in the infants, profoundly alters microbiota composition and diversity [40,41]. Antibiotics can deplete beneficial groups such as *Actinobacteria* and *Bacteroidetes* while favouring the expansion of potentially pathogenic taxa including *Proteobacteria*, *Enterococcus*, and *Klebsiella* [42,43]. These disruptions not only reduce overall microbial biodiversity but are also associated with long- term health disorders [44,45].

Antibiotic exposure is strongly associated with diarrhoea [46]. Among the clinical manifestations of early-life dysbiosis, AAD is one of the most frequent and clinically relevant outcomes. It is definite as the occurrence of three or more loose stools within a 24 h period following antibiotic administration. This condition can manifest within hours or up to eight weeks after the start of antibiotic therapy [44]. Its incidence in children ranges from 11% to over 40%, depending on antibiotic class, treatment duration, and host-related factors [47]. These findings highlight the vulnerability of the infant microbiome during this critical window, underscoring the need for careful stewardship of antibiotic use and the potential role of microbiota-supporting interventions such as probiotics, prebiotics, synbiotics, postbiotics, and paraprobiotics [48,49]. These recommendations are supported by the most recent international paediatric guidelines, including the 2023 ESPGHAN/European Society for Paediatric Infectious Diseases (ESPID) guidance on acute gastroenteritis and antibiotic-associated diarrhoea as well as the 2023 World Gastroenterology Organisation (WGO) Global Guidelines on Probiotics and Prebiotics, both of which explicitly list the strain *S. boulardii* among the evidence-based probiotics recommended for children [50,51]. Importantly, *S. boulardii* represents the only yeast included among the probiotics recommended by current international guidelines in this setting. Clinical evidence further supports the role of probiotics in reducing the risk and severity of AAD, respiratory tract infections, and allergic manifestations in children [38].

Among these, *S. boulardii* is specifically recommended for the prevention of AAD in both hospital and outpatient settings, when administered at a daily dose of at least 5 × 10^9^ Colony-Forming Units (CFU), starting at the initiation of antibiotic therapy [52,53]. Systematic reviews and meta-analyses of clinical trials have confirmed the strain-specific efficacy of *S. boulardii*. McFarland et al. analysed 22 randomised controlled trials and observed a significant reduction in AAD with *S. boulardii* [30]. Abidi corroborated these findings in 300 Indian children, reporting significantly fewer diarrheal episodes in the probiotic group compared with those receiving antibiotics alone [54]. Szajewska et al. synthesized data from 21 randomized controlled trials, demonstrating that the risk of AAD decreased from 20.9% to 8.8% in children treated with *S. boulardii* [52]. Similarly, another review including 21 studies reported substantially lower rates of diarrhoea in the probiotic group [27].

More recent evidence suggests that both *S. boulardii* and *Lactobacillus rhamnosus GG* are among the most effective single-strain probiotics for preventing paediatric AAD [20,55]. Furthermore, a large body of clinical research, supported by systematic reviews and randomized controlled trials, demonstrates the efficacy of *S. boulardii* in PAGE. Szajewska et al. demonstrated shorter diarrhoea duration, reduced stool frequency, and decreased hospitalisation [52], findings corroborated by Padayachee et al. and Fu et al. [56,57]. Meta-analyses consistently report that *S. boulardii* is safe in children, with only rare cases of fungaemia reported in severely immunocompromised individuals [52,56,57]. It is important to note that most of the cited clinical evidence has been generated using the well-characterised strain *S. boulardii.*

## 4. Adult Dysbiosis and the Role of *Saccharomyces boulardii*

By three years of age, the gut microbiota reaches an adult-like composition, characterized by the dominance of *Firmicutes, Bacteroidetes*, and *Actinobacteria* [58]. In adulthood, it typically exhibits stability and resilience, although its composition can vary among individuals and within the same individual, influenced by factors such as age and medication use [59]. A healthy adult microbiota is characterised by significant taxonomic diversity, high gene richness, and a core community that supports metabolic functions, immune regulation, and protection against pathogens [58]. As individuals age, immunosenescence and dietary changes contribute to microbial shifts, including an increase in *Clostridia* and *Proteobacteria* and a reduction in *Bifidobacterium*, a decline associated with elevated inflammation [60]. These alterations have been correlated with a broad spectrum of diseases, including cardiovascular, metabolic, inflammatory, oncological, respiratory, hepatic, and renal disorders [59,61,62]. In summary, the adult gut microbiota operates as an intricate, resilient ecosystem that remains susceptible to external disturbances, with its disruption representing a significant risk factor across multiple disease domains. Meta-analyses of randomised controlled trials demonstrate that *S. boulardii* significantly reduces the risk of AAD, compared with control or placebo groups, with moderate effect sizes [30,52,53]. These findings are aligned with the 2023 WGO Global Guidelines on Probiotics and Prebiotics, which list *S. boulardii* among the evidence-based options for the prevention of antibiotic-associated diarrhoea in adults [51].

Beyond its clinical efficacy in reducing AAD, supplementation with *S. boulardii* CNCM I-745 has also been shown to exert beneficial effects on gut microbiota composition and recovery following antibiotic exposure. Probiotic co-administration during and after amoxicillin–clavulanate therapy significantly modulates bacterial communities, increasing α-diversity, promoting recovery of commensal families (e.g., *Lachnospiraceae, Ruminococcaceae, Akkermansiaceae*), and limiting the expansion of opportunistic taxa induced by antibiotics (*Enterobacteriaceae*, *Enterococcaceae*, *Peptostreptococcaceae*, *Tannerellaceae*) [63]. Consistent with these findings, a randomized controlled study in 49 healthy volunteers demonstrated that intake of *S. boulardii* attenuated antibiotic-induced microbiota alterations, restricted *Escherichia* overgrowth, and significantly reduced AAD, with adverse events reported in only 16.7% of subjects compared with 50% receiving antibiotics alone [64]. Similarly, in a prospective study using high-resolution molecular analysis in 60 women treated for bacterial vaginosis, concomitant or subsequent *S. boulardii* administration mitigated the one-log reduction in bacterial biomass and persistent diversity loss induced by ciprofloxacin plus metronidazole, preserving major bacterial groups, accelerating microbial recovery, and restoring individual microbiota profiles in ~88% of probiotic-treated subjects [65].

Building on this evidence of microbiota modulation and improved tolerability, the adjunctive role of *S. boulardii* CNCM I-745 has also been investigated in the setting of *Helicobacter pylori* eradication therapy, particularly in light of the limitations associated with current regimens. Eradication of *H. pylori* relies on antibiotic-based triple or quadruple regimens; however, increasing antimicrobial resistance and frequent gastrointestinal adverse events are progressively compromising treatment effectiveness and tolerability [66,67,68,69]. In this regard, WGO Global Guidelines refer to the use of selected probiotic formulations, including *S. boulardii*, as adjunctive options during *H. pylori* eradication therapy to reduce gastrointestinal adverse events and improve treatment tolerability [51]. Within the context of AAD, the adjunctive use of *S. boulardii* CNCM I-745 during *H. pylori* eradication therapy has been investigated, particularly in light of the limitations of current regimens. Standard eradication relies on antibiotic-based triple or quadruple therapies; however, rising antimicrobial resistance and frequent gastrointestinal adverse events increasingly compromise both efficacy and tolerability [66,67,68,69].

In this setting, the WGO Global Guidelines highlight selected probiotic formulations, including *S. boulardii*, as adjunctive options to reduce gastrointestinal adverse events and enhance treatment tolerability [51]. Recent evidence supports the beneficial role of *S. boulardii* in *H. pylori* eradication. A meta-analysis of 19 randomized trials (5036 patients) showed an ~11% relative increase in eradication rates and a ~50% reduction in gastrointestinal adverse events, with diarrhoea risk lowered by ~60–65% [66]. Consistently, a randomized trial in 144 *H. pylori*-positive adults with non-ulcer dyspepsia found that adding *S. boulardii* CNCM I-745 to triple therapy improved eradication (75% vs. 65%) and reduced adverse events (18.7% vs. 45.8%), particularly diarrhoea, while bismuth-based quadruple therapy achieved 93% eradication [67]. Similarly, a prospective randomized open-label study in 199 adults reported that supplementation with *S. boulardii* CNCM I-745 during standard sequential therapy increased eradication by ~11%, reduced overall gastrointestinal adverse events by ~39% (including a ~44% reduction in AAD), and improved treatment adherence by ~4% [68]. Several complementary mechanisms may underlie the beneficial effects of *S. boulardii* CNCM I-745 during *H. pylori* eradication. Supplementation has been linked to a reduced abundance of antimicrobial resistance genes compared with standard triple therapy, suggesting a role in limiting antibiotic-driven selection and expansion of resistance determinants [69]. Moreover, *S. boulardii* appears to modulate host immune responses by lowering bacterial burden and gastric lymphoid follicle formation, enhancing mucosal IgA secretion and antimicrobial peptide production, and downregulating pro-inflammatory signalling pathways, thereby attenuating infection-associated mucosal inflammation [70]. Finally, its use has been associated with a more balanced gastric microbiota, characterized by modulation of anaerobic bacterial populations and enrichment of beneficial commensals, particularly *Lactobacillus* species [71].

Notably, *S. boulardii* CNCM I-745 is the only yeast strain included among the probiotic species currently recommended by international guidelines. In adults, clinical evidence supports the use of *S. boulardii* primarily for the prevention of AAD, with additional evidence for its role in diarrhoea caused by Clostridioides difficile infection and in the management of travellers’ diarrhoea. Typical therapeutic regimens involve administration of 250 mg twice daily, initiated on the first day of antibiotic therapy and continued throughout the course, occasionally extending for a few days thereafter [30,52,53].

## 5. Perspectives

This narrative review reflects the shared perspective of a multidisciplinary panel of experts, aiming to critically assess the current evidence on the use of *S. boulardii* in both paediatric and adult populations. *S. boulardii* is a unique probiotic yeast with well-documented antimicrobial, antitoxin, barrier-enhancing, and immunomodulatory properties. Traditionally, its use has been limited to the short-term management of acute diarrhoea, particularly in the context of gastroenteritis or antibiotic therapy. Nonetheless, the available evidence, combined with extensive real-world clinical use, indicates that *S. boulardii* is a safe and effective tool not only for short-term symptom management but also for the broader, long-term goal of restoring gut microbiota resilience in both children and adults. Diarrhoea should not be regarded merely as a transient symptom, but as a sentinel marker of gut dysbiosis. Its timely prevention and treatment are therefore essential, not only to relieve clinical discomfort but also to protect microbiota resilience and reduce the risk of long-term complications. In primary care, efforts should be directed towards preventing antibiotic-associated diarrhoea and supporting microbiota resilience through lifestyle, nutritional, and biological measures. In this context, timely administration, at the initiation of antibiotic treatment or within the first 48 h of an adequately dosed, clinically validated probiotic such as *S. boulardii* has been shown to prevent or reduce AAD and its underlying dysbiosis, thereby promoting a faster return to a resilient, pre-antibiotic state. Clinical evidence indicates that *S. boulardii* CNCM I-745, when administered as an adjunct to standard *H. pylori* eradication regimens, may lower the incidence of overall adverse reactions and diarrhoea. Such effects appear to enhance treatment tolerability and translate into improved patient-reported outcomes. In addition, to acute diarrhoeal episodes the most evident manifestation of gut dysbiosis, *S. boulardii* may also contribute to the relief of milder, chronic symptoms commonly associated with microbial imbalance, such as abdominal bloating, flatulence, irregular bowel habits, and a sense of incomplete digestion. These symptoms often reflect underlying dysbiosis and can significantly impair quality of life even in the absence of overt disease. Through its modulatory effects on microbial composition, intestinal permeability, and mucosal inflammation, *S. boulardii* supports the restoration of a balanced and functionally resilient gut ecosystem, thus addressing both the acute and chronic clinical consequences of dysbiosis. Therefore, beyond short-term interventions, *S. boulardii* may be employed in extended and cyclic regimens aimed at supporting long-term microbiota recovery.

In children, a daily dose of 250 mg is commonly used, whereas in adults 250 mg twice daily is recommended, typically for 10 days per month over a total period of 3–4 months. To optimise outcomes, probiotic therapy should be complemented by healthy dietary habits and regular physical activity. It should be acknowledged that the current body of evidence on *S. boulardii* has inherent limitations. Most available evidence on *S. boulardii* derives from trials assessing clinical outcomes such as antibiotic-associated diarrhoea and acute gastroenteritis, which are recognised markers of dysbiosis. However, relatively few studies have directly evaluated dysbiosis as a primary endpoint through microbiota analyses. In addition, the proposed cyclic dosing regimens are based on clinical practice and expert opinion rather than large randomized controlled trials. Finally, although *S. boulardii* is generally recognised as safe in both children and adults, rare cases of fungaemia have been reported in severely ill or immunocompromised patients, underscoring the importance of careful patient selection and risk assessment in these settings.

## 6. Conclusions

The gut microbiome, in both children and adults, plays a pivotal role in health and disease. It is shaped by internal factors such as genetics, immune function, and ageing, as well as external influences including diet, medications, and environmental exposures. When the balance of this ecosystem is disrupted—a state known as dysbiosis—the effects can extend beyond the digestive tract, influencing immunity, inflammation, and metabolism. Dysbiosis has also been linked to systemic consequences such as disrupted gut–brain communication, greater vulnerability to disorders of energy balance, and an increased risk of chronic inflammatory conditions. Although the strength of these associations varies across different conditions and causal links remain partly unclear, growing evidence highlights the importance of dysbiosis in the onset and progression of many diseases. This knowledge supports strategies that go beyond symptom control, aiming instead to restore microbiota composition and function. Diarrhoea, particularly when linked to antibiotic use, is often an early sign of microbiota disruption. Timely interventions, such as probiotic supplementation, can help prevent or reduce this complication.

Among available probiotics, *Saccharomyces boulardii* CNCM I-745 stands out for its unique biological properties and consistent evidence of safety and efficacy in both children and adults. It is well established in preventing and managing antibiotic-associated diarrhoea and has also shown promise as an adjunct to standard *Helicobacter pylori* eradication therapies. However, while *Saccharomyces boulardii* is encouraging, current data do not yet support its effectiveness across all dysbiosis-related conditions. More robust randomized controlled trials, with clinically relevant outcomes and longer follow-up, are needed to clarify its role in complex scenarios. Overall, an evidence-based use of *Saccharomyces boulardii* CNCM I-745 may be considered as part of broader strategies to strengthen microbiota resilience and promote gastrointestinal health in children and adults.

## Data Availability

No new data were created or analyzed in this study. Data sharing is not applicable to this article.

## Abbreviations

The following abbreviations are used in this manuscript:

AAD	Antibiotic-associated diarrhoea
CFU	Colony-Forming Units
ESPGHAN	European Society for Paediatric Gastroenterology, Hepatology and Nutrition
ESPID	European Society for Paediatric Infectious Diseases
FAO/WHO	Food and Agriculture Organization of the United Nations/World Health Organization
*H. pylori*	*Helicobacter pylori*
PAGE	Pediatric Acute Gastroenteritis
SCFAs	Short-chain fatty acids
*S. boulardii*	*Saccharomyces boulardii* CNCM I-745
WGO	World Gastroenterology Organisation
